# Altered cortical processing of somatosensory input in pre-term infants who had high-grade germinal matrix-intraventricular haemorrhage

**DOI:** 10.1016/j.nicl.2019.102095

**Published:** 2019-11-28

**Authors:** Kimberley Whitehead, Laura Jones, Maria Pureza Laudiano-Dray, Judith Meek, Lorenzo Fabrizi

**Affiliations:** a Department of Neuroscience, Physiology and Pharmacology, University College London, London WC1E 6BT, United Kingdom; b Elizabeth Garrett Anderson Wing, University College London Hospitals, London WC1E 6BD, United Kingdom

**Keywords:** Premature, Lesion, Tactile, Sensory, Evoked potential, Event-related potential

## Abstract

High-grade (large) germinal matrix-intraventricular haemorrhage (GM-IVH) is one of the most common causes of somatomotor neurodisability in pre-term infants. GM-IVH presents during the first postnatal week and can impinge on somatosensory circuits resulting in aberrant somatosensory cortical events straight after injury. Subsequently, somatosensory circuits undergo significant plastic changes, sometimes allowing the reinstatement of a somatosensory cortical response. However, it is not known whether this restructuring results in a full recovery of somatosensory functions. To investigate this, we compared somatosensory responses to mechanical stimulation measured with 18-channels EEG between infants who had high-grade GM-IVH (with ventricular dilatation and/or intraparenchymal lesion; *n* = 7 studies from 6 infants; mean corrected gestational age = 33 weeks; mean postnatal age = 56 days) and age-matched controls (*n* = 9 studies from 8 infants; mean corrected gestational age = 32 weeks; mean postnatal age = 36 days). We showed that infants who had high-grade GM-IVH did not recruit the same cortical source configuration following stimulation of the foot, but their response to stimulation of the hand resembled that of controls. These results show that somatosensory cortical circuits are reinstated in infants who had GM-IVH, during the several weeks after injury, but remain different from those of infants without brain injury. An important next step will be to investigate whether these evidences of neural reorganisation predict neurodevelopmental outcome.

## Highlights

• Infants who had GM-IVH recruit different cortical sources following foot stimulation.
• Results indicate restructuring of somatosensory processing during the weeks after GM-IVH.
• GM-IVH is more detrimental for lower than upper limb somatosensory processing.

## 1 Introduction

Intraventricular haemorrhage arises from the germinal matrix (GM-IVH) – a highly vascularised transient structure of the pre-term brain (de Vries, 2018). GM-IVH presents during the first postnatal week and its cause is unclear, although it is associated with respiratory distress and cardiovascular problems and most frequent in extremely pre-term infants (Ancel et al., 2015; Chalak et al., 2011; Levene et al., 1982).

High-grade (large) GM-IVH is associated with higher risk of adverse somatomotor development (Ancel et al., 2006; Payne et al., 2013), likely due to its disruption of motor and somatosensory circuits. During the week straight after injury, the resultant compression and acute inflammation of periventricular tissue (Adler et al., 2010) is often reflected in the absence or gross delay of a somatosensory cortical event (Pierrat et al., 1997; Pike et al., 1997; Pike and Marlow, 2000). A response can then be reinstated during the subsequent weeks (Klimach and Cooke, 1988; Pierrat et al., 1997; Pike and Marlow, 2000; Slater et al., 2010; Vries et al., 1990), which is associated with positive neurodevelopmental outcome (Willis et al., 1989). The return of a response likely represents the ability of thalamo-cortical tracts to adopt alternative trajectories to circumvent the injury and reach the cortex (Arichi et al., 2014; Guzzetta et al., 2007; Staudt et al., 2006; Wilke et al., 2009).

However, processing of somatosensory input in pre-term infants involves multiple steps, beyond the simple transmission of the information to the brain (Donadio et al., 2018; Hrbek et al., 1973, 1968; Pike et al., 1997; Whitehead et al., 2019). Here we hypothesised that even if plastic changes allow the reinstatement of a cortical response to somatosensory input after GM-IVH, this response could still differ from that of infants who never experienced an injury. To test this hypothesis, we compared temporal and spatial differences in somatosensory cortical events following mechanical stimulation of the foot and hand between infants who had high grade GM-IVH and age-matched controls.

## 2 Material and methods

### 2.1 Subjects

Subjects were recruited from the neonatal unit at the Elizabeth Garrett Anderson wing of University College London Hospitals. Ethical approval was obtained from the NHS Research Ethics Committee, and informed written parental consent was obtained prior to each study. Additional written parental consent was obtained to publish video data from one infant. The study conformed to the standards set by the Declaration of Helsinki. No neonates were acutely unwell at the time of study, required mechanical ventilation, or were receiving anti-seizure medication. Exclusion criteria included congenital abnormalities and severe intra-uterine growth restriction (defined here as abnormal antenatal Doppler ultrasound imaging and <2nd birth weight centile).

Subjects included six infants who had high-grade GM-IVH and eight controls without high-grade GM-IVH matched for both corrected gestational age (CGA) and postnatal age (PNA) (Table 1). The presence of GM-IVH was assessed by reviewing the reports of routine (clinically required) cranial ultrasound scans carried out during the postnatal period, all evaluated and verified by a consultant neonatologist, recorded in the infant's hospital notes. High-grade GM-IVH comprised: GM-IVH with ventricular dilatation (grade III) or GM-IVH with secondary intraparenchymal lesion (IPL) (de Vries, 2018)) (Table 1). It is typical to combine these two subcategories in a single group as high-grade GM-IVH (e.g. (Ancel et al., 2015; Chalak et al., 2011; Nevalainen et al., 2015; Olischar et al., 2007)). Control infants were defined as having either normal cranial ultrasound imaging or a small (grade I) germinal matrix haemorrhage which had not bled into the ventricles (one infant). Small germinal matrix haemorrhage is of no clinical significance (Payne et al., 2013; Radic et al., 2015) and associated with normal somatosensory cortical events during the neonatal period (Pierrat et al., 1997; Pike et al., 1997). Throughout the following text, for brevity, GM-IVH refers to high-grade GM-IVH. No infants received opiate or sedative medications in the 24 h prior to the study apart from two infants in the GM-IVH group who were respectively receiving a weaning regime of oral morphine (32 mcg/kg/dose) and chloral hydrate (50mg/kg/dose). Morphine does not affect ongoing or sensory-evoked brain activity at this age, when other medications and clinical factors are corrected for (Bell et al., 1993; Dix et al., 2018; Hartley et al., 2018). In children, chloral hydrate does not consistently affect electrical brain activity (Thoresen et al., 1997).

**Table 1:** Clinical data of infants.

	High-grade GM-IVH	Controls (No high-grade GM-IVH)
No. of EEG studies	7	9
No. of neonates	6^a^ (3 female)	8^b^ (2 female)
No. of foot stimulation trains (Right: Left)	10 (7:3)	12 (7:5)
No. of hand stimulation trains (Right: Left)	5 (3:2)	8 (3:5)
Mean (range) CGA at study (weeks+days)	33+4 (30+3-35+6)	32+3 (29+1-35+5)
Mean (range) GA at birth (weeks+days)	25+4 (23+6-30+0)	27+3 (24+5-32+4)
Mean (range) PNA at study (days)	56 (31-77)	36 (21-65)
GM-IVH details (infants ordered by ascending CGA) (limbs stimulated)	#1 L grade III / R grade III (RF, LF, RH, LH)	
	#2 L grade III / R grade III; post-haemorrhagic hydrocephalus managed by therapeutic LPs^c^ (RF, RH)	
	#3^a^ L IPL / R grade II (first study: RF, LF; second study: RF)	
	#4 L grade III / R grade III (RF, RH)	
	#5 L grade I / R IPL (RF, LF)	
	#6 L grade I / R IPL; post-haemorrhagic hydrocephalus managed by therapeutic LPs^c^ (RF, LH)	

PNA indicates postnatal age; GA indicates gestational age at birth; CGA indicates GA + PNA

LPs = lumbar punctures; L = left; R = right; F = foot; H = hand

a One infant (#3) studied twice with an inter-study interval of 14 days.

b One infant studied twice with an inter-study interval of 27 days.

c Managed post-haemorrhagic hydrocephalus is not consistently associated with additional somatosensory or other sensory cortical dysfunction, beyond that associated with the GM-IVH (Klebermass-Schrehof et al., 2013; Pierrat et al., 1997; Radic et al., 2015), although appears to confer a small degree of additional neurodevelopmental risk (Brouwer et al., 2008).

### 2.2 EEG recording

Up to eighteen recording electrodes (disposable Ag/AgCl cup electrodes) were positioned according to the modified international 10/10 electrode placement system. Montages customarily included Cz, CPz, C3, C4, CP3, CP4, F3, F4, F7, F8, T7, T8, P7, P8, O1, O2, and sometimes additionally Fz, P3, P4, POz, Oz, TP9, TP10. Two infants were studied twice ≥14 days apart which does not underestimate the variance (see the supplemental information in Fabrizi et al. (2011)), leading to a total of 16 EEG studies. A reduced number of electrodes were applied if the infant became unsettled during set-up (median 18 electrodes applied; 14/16 EEG studies included ≥15 electrodes, minimum 10; no statistically significant difference in the number of electrodes applied between groups: *p* = =.258, unpaired t test). The reference electrode was placed at either Fz or FCz. Target impedance of electrodes was <10 kΩ (André et al., 2010). A single lead I ECG was recorded from both shoulders. Respiratory movement was monitored with an abdominal movement transducer. EEG was recorded with a direct current (DC)-coupled amplifier from DC-800 Hz using the Neuroscan (Scan 4.3) SynAmps2 EEG/EP recording system. Signals were digitized with a sampling rate of 2 kHz and a resolution of 24 bit.

### 2.3 Tactile stimulation

Mechanical taps were delivered to the lateral edge of the infants’ palms and heels using a hand-held tendon hammer with a 15mm^2^ contact surface (see Supplementary Video). The hammer had a piezo-electric transducer that allowed to record the precise timing of the stimulation on the EEG recording (Worley et al., 2012). The interstimulus interval was long, variable, and self-paced by the experimenter (average 15 s) as shorter intervals could attenuate the somatosensory response (Desmedt and Manil, 1970; Gibson et al., 1992; Nevalainen et al., 2015; Stjerna et al., 2012). In case the infant moved, the tap was delayed for several seconds to avoid potential modulation of the somatosensory response by the movement (Saby et al., 2016) and to allow movement artefacts to resolve. It was not possible to stimulate one of the two hands when a cannula or longline (peripherally inserted central venous catheter) was present, and a reduced number of stimuli were delivered if the infant became unsettled. This resulted in a total of 35 stimulation trains (i.e. stimulated limbs) of 2-32 stimuli (mean 13) delivered to one of the four limbs. There was no statistically significant difference in the distribution of right and left limbs stimulated between the GM-IVH and control groups (feet: *p* = =.571, hands: *p* = =.429, chi-squared tests; Table 1).

**Supplementary Video: Tactile stimulation of the heel in subject #6.** Vertical yellow line indicates the occurrence of a tap. To maintain the infant's comfort, they remain wrapped in their bedding, with only a small amount of skin uncovered to deliver the tap. Data are displayed referred to Fz (acquisition montage) and with a bandpass filter of 0.5–70 Hz. Scale bar bottom left hand corner. Solid grey vertical lines mark each second and dashed grey vertical lines mark each 200 milliseconds.

### 2.4 Analysis: pre-processing

**Fig. 1:**
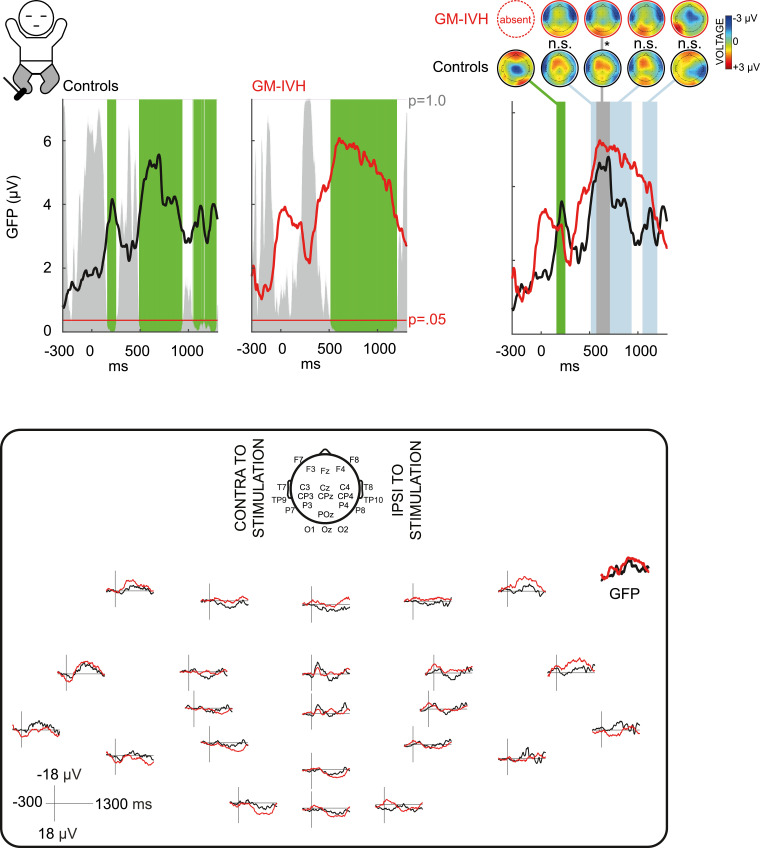
**Brain activity in response to foot stimulation is different in infants who had germinal matrix-intraventricular haemorrhage (GM-IVH).** Upper panel left: grand average global field power (GFP) showing consistent neuronal activation in controls and in infants who had GM-IVH (somatosensory cortical events, green shading, the height of the grey area indicates the p-value of the Topographic Consistency Test). Upper panel right: topoplots display mean topographies across time points of intra-group topographic consistency (green shading) and time points in which both groups had consistent neuronal activation of the same topography (blue shading) or different topography (grey shading) (normalised by GFP). Lower panel: Grand average of the EEG recordings and GFP. 2-column fitting image. (For interpretation of the references to color in this figure legend, the reader is referred to the web version of this article.)

**Fig. 2:**
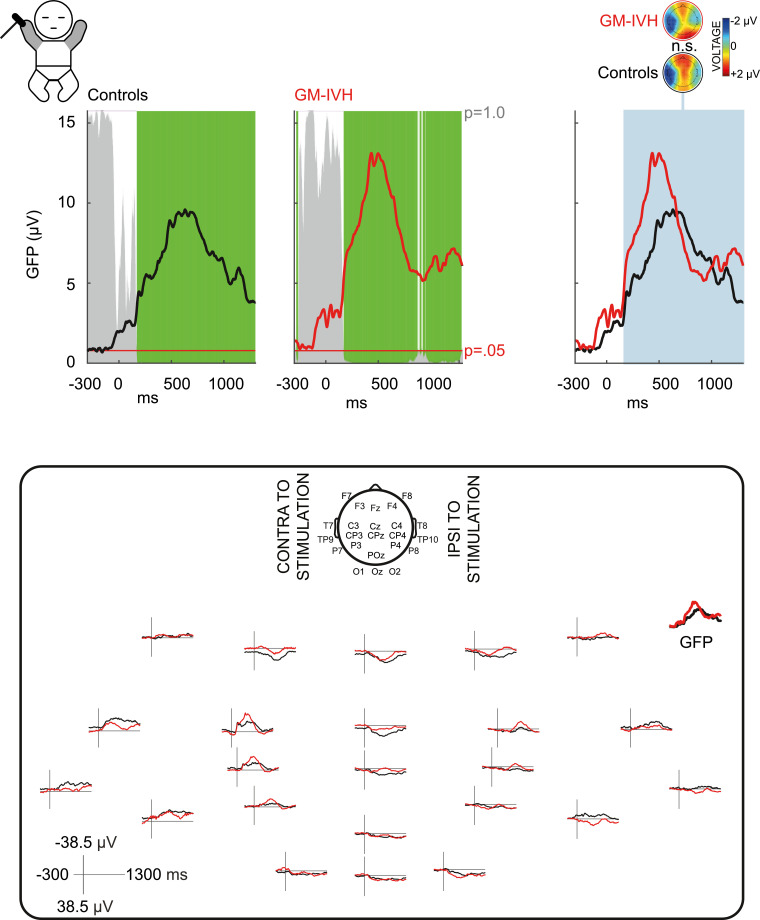
**Brain activity in response to hand stimulation is not significantly different in infants who had germinal matrix-intraventricular haemorrhage (GM-IVH).** Upper panel left: grand average global field power (GFP) showing consistent neuronal activation in controls and in infants who had GM-IVH (somatosensory cortical events, green shading, the height of the grey area indicates the *p*-value of the Topographic Consistency Test). Upper panel right: topoplots display mean topographies across time points in which both groups had consistent neuronal activation of the same topography (blue shading) (normalised by GFP). Lower panel: Grand average of the EEG recordings and GFP. 2-column fitting image. (For interpretation of the references to color in this figure legend, the reader is referred to the web version of this article.)

Data pre-processing was carried out using EEGLAB v.14 (Swartz Center for Computational Neuroscience). Data were re-referenced to common average (retrieving the reference channel Fz or FCz). Four trials from three datasets containing artefact were completely discarded which resulted in a total of 436 trials being analysed. There was no statistically significant difference in the number of trials analysed per stimulation train between the GM-IVH and control groups (hands: *p* = =.081, feet: *p* = =.371, unpaired t tests). Up to two bad channels (poor contact with the scalp) from two datasets were removed. Missing and discarded recordings were estimated with spherical interpolation as implemented in EEGLAB. Data were bandpass filtered at 0.5–40 Hz (2nd order Butterworth filter) with a 50 Hz notch filter (4th order Butterworth filter) and then epoched from -300 until +1300 ms around the stimulus. All EEG epochs were baseline corrected by subtracting the mean baseline signal and averaged across repetitions (i.e. single average response per limb stimulated). Traces from each stimulation train were aligned to correct for intra-subject latency jitter with Woody filtering (Woody, 1967) (alignment window: 160–210 ms at midline central (foot stimulation) or contralateral central (hand stimulation) electrode; maximum allowed jitter -40 to +40 ms). The degree to which trials were aligned did not statistically differ between groups (feet: mean 20 (SD: 13) vs. 21 (SD: 13) ms, and hands: mean 19 (SD: 14) vs. 18 (SD: 12) ms, for GM-IVH vs. controls respectively (*p* ≥ .450, unpaired t tests). EEG recordings following stimulation of the left hemi-body were ‘side-swapped’ so that recordings contralateral to the stimulation site appear on the ‘same side’ of the scalp (left in Figs. 1 and 2, which is then labelled as ‘contralateral to stimulation’).

### 2.5 Analysis: intra-group characterisation of somatosensory cortical response

Data analysis was carried out using Ragu (Koenig et al., 2011), which identifies the presence of, and then inter-group differences between, somatosensory cortical events using non-parametric permutation statistics timepoint by timepoint (n = 1000 randomization runs among channels). The presence and timing of somatosensory cortical events was established separately for infants with and without GM-IVH using the topographic consistency test (Koenig and Melie-García, 2010). This test examines if and at what latencies a stimulus consistently elicits the same scalp field distribution across subjects using Global Field Power (GFP) measurements (Koenig and Melie-García, 2010). To account for multiple comparisons, the presence of somatosensory cortical events was considered significant if the time period in which the test resulted in p < .05 was at least 5% of the analysis window. Unlike methods to control for multiple comparisons such as false discovery rate, this considers the probability that consecutive samples differ (Guthrie and Buchwald, 1991).

### 2.6 Analysis: inter-group comparison of somatosensory cortical response

If the stimulus elicited somatosensory cortical events at the same latencies for the two groups, the neuronal activation could still differ in source configuration or magnitude between the groups. To test for inter-group differences in source configuration, we compared scalp field maps normalised to their GFP (i.e. magnitude independent, ‘topographic analysis of variance’ (TANOVA) (Wirth et al., 2008)) for those periods when both groups had a somatosensory cortical event. To test for differences in magnitude, we compared GFP for those periods when both groups had a somatosensory cortical event which did not differ significantly in source configuration (Habermann et al., 2018). For display purposes we plotted the GFP of the grand average EEG response for each group (Figs. 1 and 2 and Supplementary Fig. 1) (the statistical comparison of GFP between groups uses each infant's GFP data).

## 3 Results

### 3.1 Results: brain activity in response to foot stimulation differed in infants who had GM-IVH

Control infants had an early cortical event (151–257 ms), with symmetrical vertex negativity, which was absent in infants who had GM-IVH (Fig. 1, Supplementary Fig. 1). The response of both groups then included a brief event starting at 487 ms (controls) and 512 ms (GM-IVH) until 566 ms with a symmetrical distribution (Fig. 1). However, the responses then diverged, with that of infants who had GM-IVH lacking the symmetrical vertex positivity observed in controls (*p* < 0.05 TANOVA for 154 ms) (Fig. 1, Supplementary Fig. 1). The responses of the two groups then converged again in topography. Between 720 and 936 ms both had a bi-centro-temporal negativity, and between 1046 and 1202 ms a contralateral positivity and ipsilateral negativity. The response in controls lasted very slightly longer than GM-IVH infants (until 1295 ms) (Fig. 1). There was no statistically significant difference in the magnitude of the response between the two groups (GFP during those periods when both groups had a cortical event *p* ≥ 0.12) (Fig. 1).

### 3.2 Results: brain activity in response to hand stimulation was similar across infants who had GM-IVH and controls

In contrast to foot stimulation, the response to hand stimulation had almost exactly the same duration in infants who had GM-IVH and controls (overlap: 167–1300 ms) (Fig. 2). Its mean topography comprised a negativity over the contralateral centro-temporal region, and positivity maximal at the midline frontal and posterior areas, and did not significantly differ between the two groups for its whole duration (*p* ≥ 0.27 TANOVA) (Fig. 2). The magnitude of the response in infants who had GM-IVH was greater than controls, but this did not reach statistical significance (*p* < 0.10 GFP 411-448 ms) (Fig. 2).

## 4 Discussion

Here we show that in pre-term infants who had GM-IVH, brain responses to mechanical stimulation of the limbs, even if recovered, are still significantly different from controls several weeks after the injury.

Consistent somatosensory activity following stimulation of the foot starts only at 512 ms in infants who had GM-IVH, 361 ms later than controls: infants who had GM-IVH lack a somatosensory event that occurs at approximately 200 ms in controls and which resembles previous reports (Cindro et al., 1985, 1985; Donadio et al., 2018; Fabrizi et al., 2011; Minami et al., 1996; Pike et al., 1997; Slater et al., 2010; Whitehead et al., 2019). This delay could arise from altered thalamo-somatosensory cortical transmission: in infants who had a large GM-IVH these tracts can be as extensively re-routed as to pass via the insula (Arichi et al., 2014), and neonatal animal models of this injury demonstrate disruption of structural thalamo-somatosensory cortical maps (Quairiaux et al., 2010). As relatively early responses with central scalp negativity in neurologically normal pre-term infants have been attributed to the primary somatosensory cortex, and their topographic organisation interpreted as reflecting the neural map of limb representation (Nevalainen et al., 2015, 2012; Whitehead et al., 2019), our data suggest that infants who had GM-IVH have impaired processing at this crucial first ‘rung’ of hierarchical sensory functioning.

When consistent somatosensory activity following foot stimulation begins in infants who had GM-IVH, they briefly share the same cortical source configuration as controls (for 54 ms) but this source configuration quickly diverges, indicating further differences in neural processing between groups. It is likely that the impaired early encoding of tactile input disrupts the trajectory of subsequent processing steps, limiting the GM-IVH infants’ ability to recruit the same hierarchical processing pathways as controls (Thivierge and Marcus, 2007; Whitehead et al., 2019). Indeed, animal models confirm that hierarchical propagation of somatosensory-evoked cortical activity depends upon the initial activation (Quairiaux et al., 2011), and a fMRI study of infants who had GM-IVH demonstrated that they were unable to recruit the supplementary motor area into their somatosensory cortical response, unlike controls (Arichi et al., 2014). Nevertheless, despite the early differences observed here between infants who had GM-IVH and controls (until 720 ms), during the latter part of the cortical response they again share the same source configuration. This could reflect the ability of restructured somatosensory circuits in infants who had GM-IVH to eventually realign the response with that observed in uninjured infants.

Failure to recruit the same cortical source configuration as controls in infants who had GM-IVH is specific to stimulation of the foot, and not the hand. Greater impairment of somatosensory processing of lower limb input, relative to the upper limbs, has been reported for the early thalamo-cortical afferent volley in GM-IVH (Pierrat et al., 1997) and is likely explained by the projections of those limbs being located closer to the ventricular wall (Staudt et al., 2000). Here we show that relatively greater differences in lower limb somatosensory functioning between infants who had GM-IVH and controls extends also to the later processing steps. All the same, following hand stimulation there was a trend for infants who had GM-IVH to have an amplified somatosensory cortical event. In line with this, comparable amplification of *background* cortical activity has been reported in neonates following brain injury, often termed ‘dysmaturity’ (Okumura et al., 2002; Watanabe et al., 1999; Whitehead et al., 2016), to reflect that large cortical events are typically associated with immature brain activity (Fabrizi et al., 2011; Hrbek et al., 1973; Milh et al., 2007; Vanhatalo et al., 2009; Whitehead et al., 2018b, 2018a). Future research should attempt to distinguish whether such amplification is potentially adaptive (Antón-Bolaños et al., 2019; Burbridge et al., 2014; Frank et al., 2001; Jha et al., 2005; Shen and Colonnese, 2016; Tolner et al., 2012; Xu et al., 2011; Zhang et al., 2012), or rather simply a marker of damage (Furlong et al., 1993; Mauguière, 2005; Riquelme and Montoya, 2010), by correlating this variable with neurodevelopmental outcome.

Although both grade III GM-IVH and IPL originate from germinal matrix haemorrhage, the exact pathophysiology of the injury will be different between that associated with compressive ischemia but no parenchymal damage (grade III) and secondary haemorrhagic venous infarction resulting in IPL (de Vries, 2018; Luo et al., 2019; Pierrat et al., 1997; Quairiaux et al., 2010; Volpe, 2009; Witte et al., 2000). Future prospective studies should examine larger populations of infants who had GM-IVH so that (i) grade III GM-IVH and IPL can be studied separately, (ii) differences in processing of somatosensory input contra- and ipsilateral to unilateral lesions can be dissociated, and (iii) inter-individual differences in alternative somatosensory cortical developmental trajectories can be delineated. Nevertheless, here we show that high-grade GM-IVH is associated with restructuring of somatosensory circuitry in the several weeks following injury resulting in inability to recruit the same cortical source configuration as controls following foot stimulation. This evidence provides insight into functional reorganisation following one of the most commonly acquired brain injuries of the pre-term period (Gale et al., 2018; Stoll et al., 2015).

## Appendix Supplementary materials

Image, application 1

Image, application 2

Supplementary Fig. 1: Individual topographies across time points in which brain activity in response to foot stimulation is different in infants who had germinal matrix-intraventricular haemorrhage (GM-IVH). Lower panel: Grand average global field power (GFP) showing time points of consistent neuronal activation (somatosensory cortical events) only in controls (green shading) and not infants who had GM-IVH, and time points in which both groups had consistent neuronal activation but of different topography (grey shading). Upper panel: Individual (within rectangles) and grand average (bold outline) topoplots display mean topographies across these time points (normalised by GFP). 2-column fitting image. Image, application 3
