# Supplementary figures and images for: Altered cortical processing of somatosensory input in pre-term infants who had high-grade germinal matrix-intraventricular haemorrhage
Source: Neuroimage Clin. 2019 Nov 28;25:102095. doi: 10.1016/j.nicl.2019.102095 (PMC6920135; doi:10.1016/j.nicl.2019.102095)

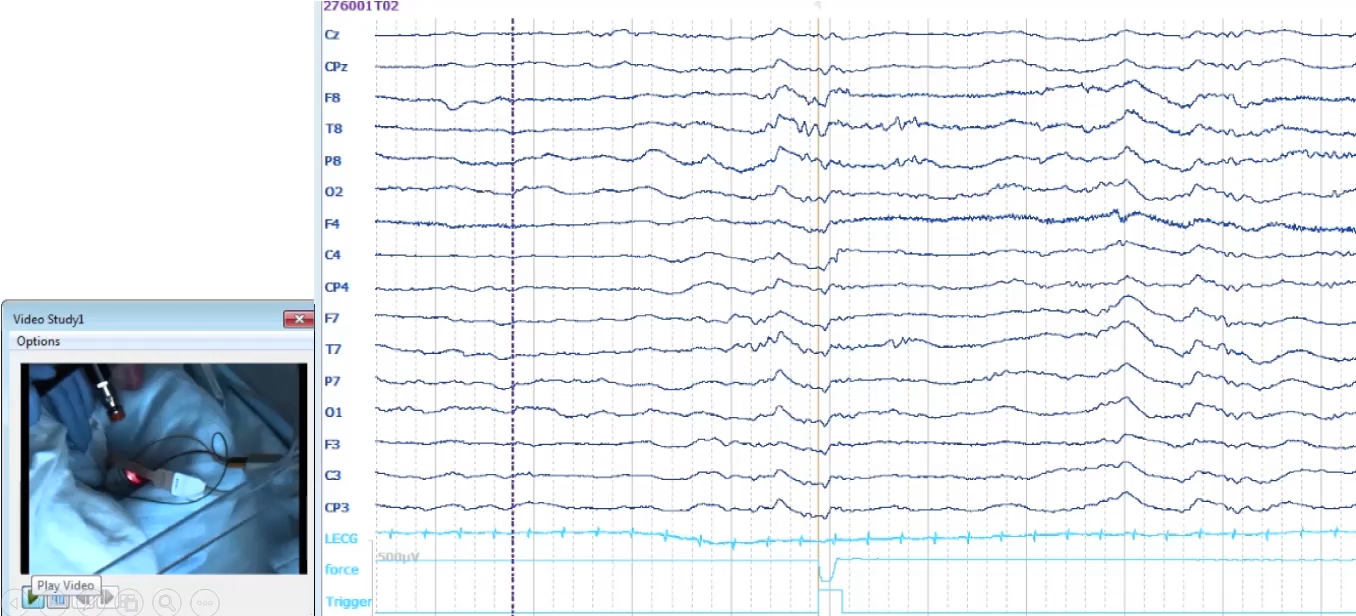

Supplement: Supplementary file 2 [file mmc2.zip › Vid1 still.tif]
